# Investigation on the Tribological Behavior and Wear Mechanism of Five Different Veneering Porcelains

**DOI:** 10.1371/journal.pone.0137566

**Published:** 2015-09-14

**Authors:** Jie Min, Qianqian Zhang, Xiaoli Qiu, Minhao Zhu, Haiyang Yu, Shanshan Gao

**Affiliations:** 1 State Key Laboratory of Oral Diseases, West China Hospital of Stomatology, Sichuan University, Chengdu, China; 2 State Key Laboratory of Traction Power, Southwest Jiaotong University, Chengdu, China; 3 Department of Stomatology, The Second People’s Hospital of Nanning City, Guangxi, China; University of Akron, UNITED STATES

## Abstract

**Objectives:**

The primary aim of this research was to investigate the wear behavior and wear mechanism of five different veneering porcelains.

**Methods:**

Five kinds of veneering porcelains were selected in this research. The surface microhardness of all the samples was measured with a microhardness tester. Wear tests were performed on a ball-on-flat PLINT fretting wear machine, with lubrication of artificial saliva at 37°C. The friction coefficients were recorded by the testing system. The microstructure features, wear volume, and damage morphologies were recorded and analyzed with a confocal laser scanning microscope and a scanning electron microscope. The wear mechanism was then elucidated.

**Results:**

The friction coefficients of the five veneering porcelains differ significantly. No significant correlation between hardness and wear volume was found for these veneering porcelains. Under lubrication of artificial saliva, the porcelain with higher leucite crystal content exhibited greater wear resistance. Additionally, leucite crystal size and distribution in glass matrix influenced wear behavior. The wear mechanisms for these porcelains were similar: abrasive wear dominates the early stage, whereas delamination was the main damage mode at the later stage. Furthermore, delamination was more prominent for porcelains with larger crystal sizes.

**Significance:**

Wear compatibility between porcelain and natural teeth is important for dental restorative materials. Investigation on crystal content, size, and distribution in glass matrix can provide insight for the selection of dental porcelains in clinical settings.

## Introduction

Porcelains have been widely used for dental restoration because of their excellent aesthetics, durability, and biocompatibility [1]. Despite these advantages, their use in clinical settings is limited by their susceptibility to fracture and excessive wear of opposing teeth [2]. Several porcelains such as feldspathic porcelain, glass-ceramics, glass-infiltrated aluminia, and zirconia are largely used as dental prosthetic materials for obtaining different restorative effects [3, 4]. Among these materials, feldspathic porcelain constitutes the outermost layer of porcelain-fused-metal restorations and usually determines the color of the dental material. Aside from fracture susceptibility, issues on friction and wear behavior of veneering porcelain are of great importance and must be addressed.

Dental restorations of porcelain fused to metal has been used in clinic for a long time. However, the wear of the opposing natural tooth or restoration is frequently observed [5]. Proper selection of dental materials is important for protecting the natural tooth [5]. At present, a specific explanation for the wear mechanisms is difficult to achieve due to the complex environments in vivo and the limitations of test methods. Studies on tooth wear caused by dental porcelains are mostly limited to the influence of surface finishing (i.e., roughness [6]) and external conditions (e.g., different loads and lubrication media [7] and various antagonist objects [8]) on abrasion resistance. Previous research reported that the mechanical property of dental porcelain was influenced by many factors, including microstructures, surface roughness, chemical composition, and mechanical features [6, 9, 10]. The material’s microstructure is identified as an important factor influencing the properties of dental restorations [9, 10]. Ho [9] suggested that the microstructure was strongly correlated with the mechanical properties of the ceramics by using CaO-P2O5 glass as additive. Jr [10] reported that the composition generated the microstructure and then effected the mechanical property of the porcelain. Those results mainly focus on the effect of the microstructure on the mechanical property of the porcelains rather than the effect on the wear resistance. However, the influence of microstructures is still unclear.

In this study, we explore the influence of the microstructure of veneering porcelains on wear mechanism under an artificial saliva environment. The overall objective is to evaluate the contribution of crystal content and size, as well as the distribution in the glass matrix of the porcelain. Findings would provide insight for the selection of dental porcelains in the clinic and the development of new dental materials in the future.

## Materials and Methods

### 2.1 Preparation of specimen

Five types of porcelains containing similar chemical components but different microstructures were used in this study (Vita master, Kiss, Vita VMK95, Vintage, and Ceramco 3). The specimens were fabricated by mixing 0.5 g of ceramic powder and 0.18 g of modeling liquid to form slurry. The slurry was poured into custom-fabricated base molds with dimensions of 20.0 mm × 10.0 mm × 2.0 mm. A firing cycle was performed using a dental porcelain furnace (Ceramco 7.0 furnace, 9495301, Dentsply Corp., USA) according to the manufacturer’s instructions. After firing, the porcelain surfaces were polished, beginning with #800 mesh SiC paper (Silicon carbide paper, Struers) and then continuing incrementally to #4000 mesh with successively finer abrasives to obtain a flat surface. Finally, the specimens were auto-glazed. Five specimens are prepared for each kind of the porcelains.

### 2.2 Hardness testing

The surface microhardness (SMH) of all the samples was performed with a microhardness tester (Duramin-1/-2; Struers, Copenhagen, Denmark) using a Knoop indenter at 300 g load for 5 s. Eight indentations spaced 20 μm from each other were made at the center of the sample surface. Specimens were etched with 2.5% hydrofluoric acid (HF) for 30 s, and then surface microstructures were observed using a scanning electron microscope (SEM) (SEM,Quanta 200,INSPECT F, Czech Republic).

### 2.3 Wear test

Wear tests were performed on a ball-on-flat PLINT fretting wear testing machine (model DS 20, Nene Corp., France) using artificial saliva as lubricant [11] at 37°C. Imposed load of 20 N and displacement amplitude of 500 μm were selected as parameters [12]. The number of cycles was changed from 100 to 500, 1000, 2000, and 10000. Al_2_O_3_ was used as antagonist. During the experiment, the friction force, cycle times, and corresponding displacement were recorded. The depth and width of wear scars were measured by a surface profiler after each test and were reconstructed in three dimensions using SPIP software 4.8.2 (Image Processing Software for Microscopy). Loss of volume was subsequently calculated. The wear surface morphologies were observed by a confocal laser scanning microscope (CLSM) (LCSM, ols1100, Olympus Corp., Japan) and SEM to explore the damage features and wear mechanism. The friction coefficient was calculated by the following equation,

$$\mu =\frac{\text{f}}{\text{F}}$$ (1)

Where μ was the friction coefficient, f was the friction force, and F was imposed load.

### 2.4 Statistical analysis

The Knoop hardness, wear volume, and wear depth of feldspathic porcelains were compared between evaluated porcelains in 10000 cycles via one-way ANOVA and least significant difference test using SPSS 17.0 (*P* = 0.05). In addition, the correlation between Knoop hardness of specimens and wear volume was analyzed via linear correlation (*r* = 0.05). A *P*-value or *r*-value of less than 0.05 was considered statistically significant.

## Results

### 3.1 Microstructure of five veneering porcelains

**Fig 1:**
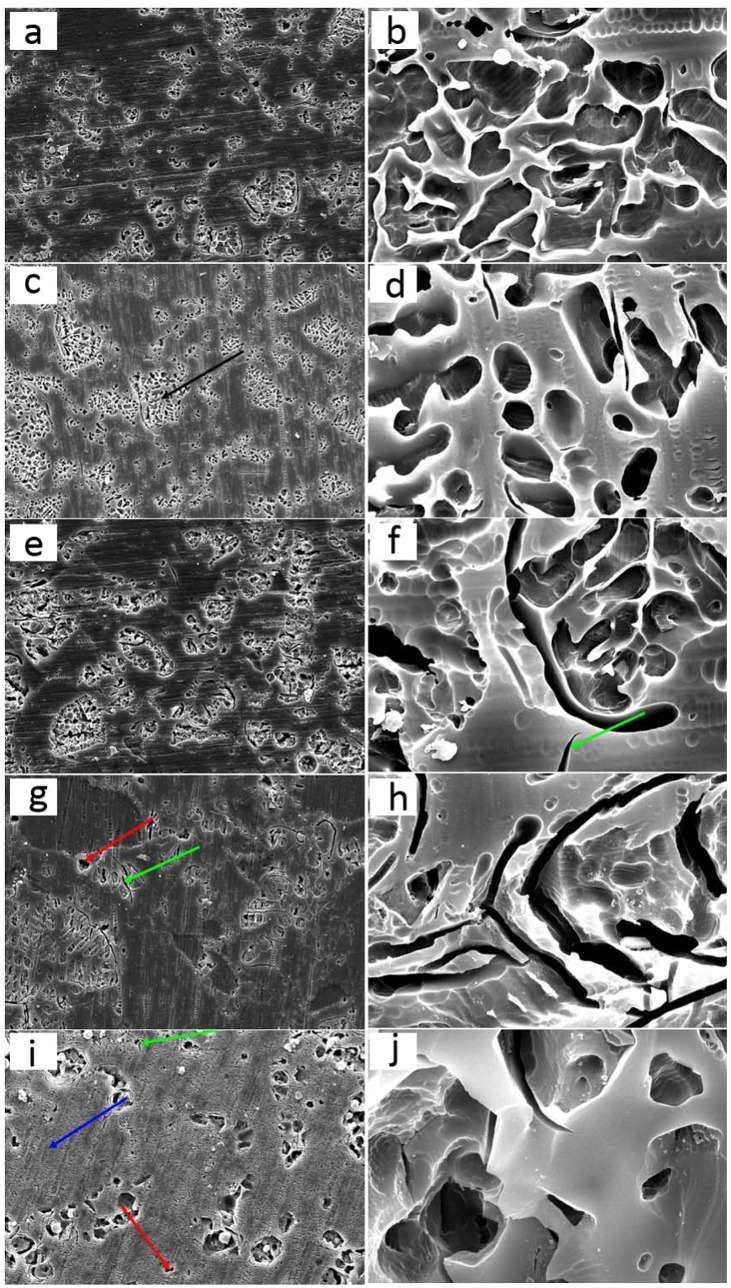
Surface micrographs of five porcelains etched with 2.5% hydrofluoric acid for 30 s. (a) Vita master, at a magnification of ×2000; (b) Vita master, at a higher magnification of ×20000; (c) Kiss, at a magnification of ×2000; (d) Kiss, at a higher magnification of ×20000; (e) Vita VMK95, at a magnification of ×2000; (f) Vita VMK95, at a higher magnification of ×20000; (g) Vintage, at a magnification of ×2000; (h) Vintage, at a higher magnification of ×20000; (i) Ceramco 3, at a magnification of ×2000; (j) Ceramco 3, at a higher magnification of ×20000.

The SEM images for the five porcelains evaluated are shown in Fig 1. All selected feldspathic porcelains are composed of a crystalline phase (Fig 1c, black arrow), a glass phase (Fig 1i, blue arrow), and porosity (Fig 1g and 1i, red arrow). The leucite crystals with different sizes, ranging from 1 μm to 10 μm, were distributed throughout the glass matrix. The most prominent difference among the five porcelains was the size of leucite crystals. For Vita master, the leucite crystals are seen in uniform distribution, with an average size of 3 μm (Fig 1a). The findings in Fig 1b show that the interfaces between the crystal and the matrix are rounded at higher magnification. In Kiss, the leucite crystals (Fig 1c and 1d) exhibit an average length of 3–5 μm and are distributed in clusters. Vita VMK95 displays greater sizes of leucite crystals (approximately 5 μm) and appear in clusters over the glass matrix (Fig 1e and 1f). The interface between the matrix and crystals is rounded which is the same as previous groups. However, microcracks (green arrow) are observed on the etched surface. Vintage (Fig 1g and 1h) reveals smaller leucite crystals of 1–3 μm, microcracks (green arrow), and porosity (red arrow). The leucite clusters appear in needle-like shapes. Finally, Ceramco 3 shows leucite crystals with average size of 5–7 μm (Fig 1i and 1j). Contrary to previous porcelain studies, the amount of leucite crystals in the glass matrix for Ceramco 3 is lower. Microcracks (green arrow) and porosity (red arrow) are also observed within the matrix. After etching, the leucite crystals were removed from the matrix as a whole, and boundaries of the matrix were exposed clearly.

### 3.2 Hardness and wear volume of five dental veneering porcelains

**Fig 2:**
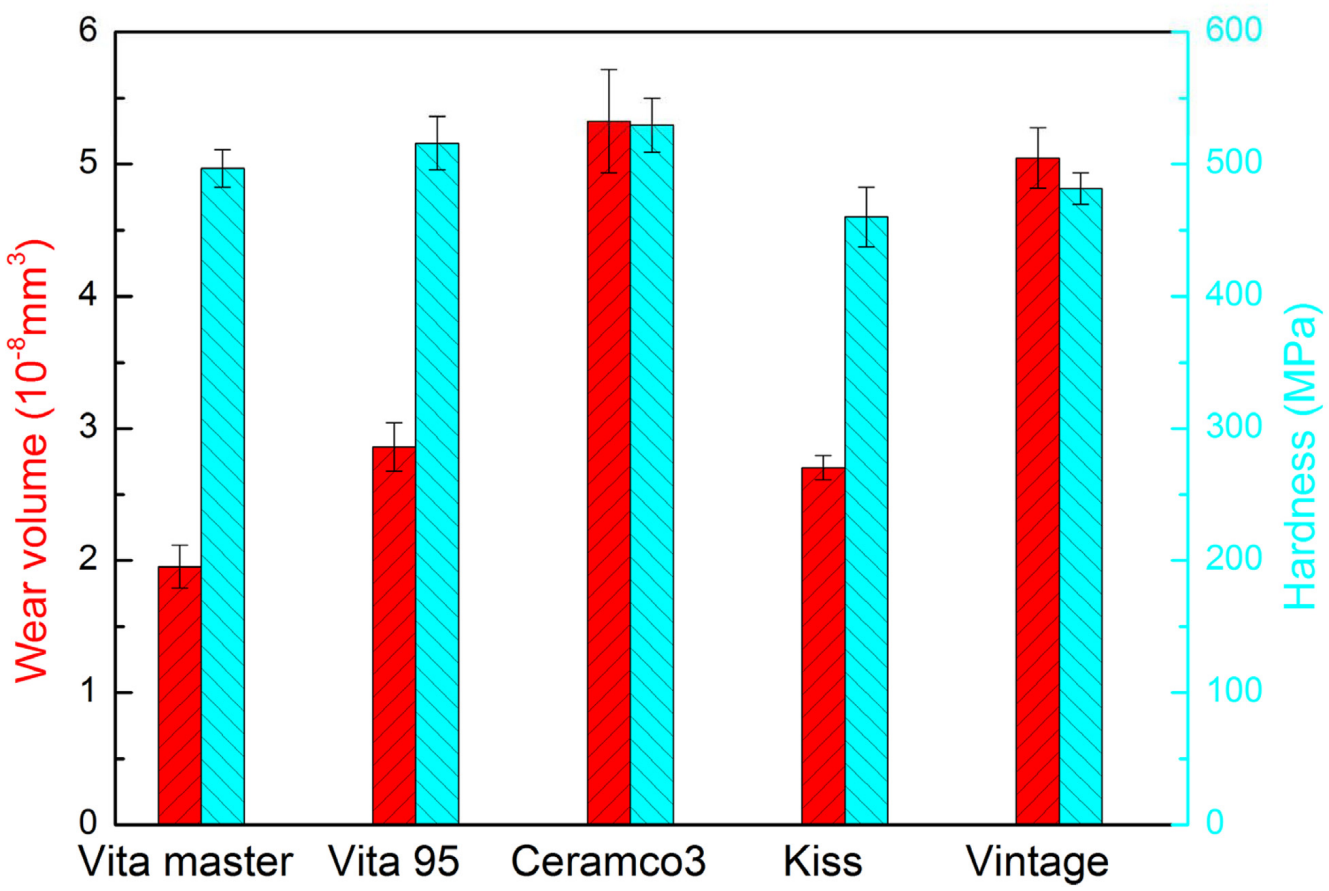
Hardness and wear volume of five dental veneering porcelains.

Indentation hardness represents a material’s resistance to deformation brought by constant compression load from a sharp object [13]. As observed in Fig 2, hardness is greatest for Ceramco 3, followed by Vita VMK95, Vita master, Vintage, and Kiss. Hardness and wear volumes of five kinds of dental veneering porcelains are shown in Table 1 and S1 Dataset. The mean wear volumes of Ceramco 3 and Vintage are almost twice that of Vita VMK95 and Kiss, whereas Vita master shows the smallest value. Vita master is the most abrasion-resistant, and Ceramco 3 is the least wear-resistant.

**Table 1:** Mean hardness and wear volume of five kinds of dental veneering porcelains.

Veneering porcelains	Hardness (HK)	Wear volume (10^-8^mm^3^)
Vita master	496.757±14.237	1.954±0.163
Vita 95	515.957±20.138	2.859±0.182
Ceramco3	529.514±20.521	5.325±0.390
Kiss	460.057±22.511	2.705±0.092
Vintage	481.557±11.921	5.047±0.228

The differences in hardness (*P* < 0.05) and in wear volume (*P* < 0.05) of the five veneering porcelains are statistically significant. However, no significant correlation between hardness and wear volume is noted (*r* = 0.2948, *P* > 0.05).

**Fig 3:**
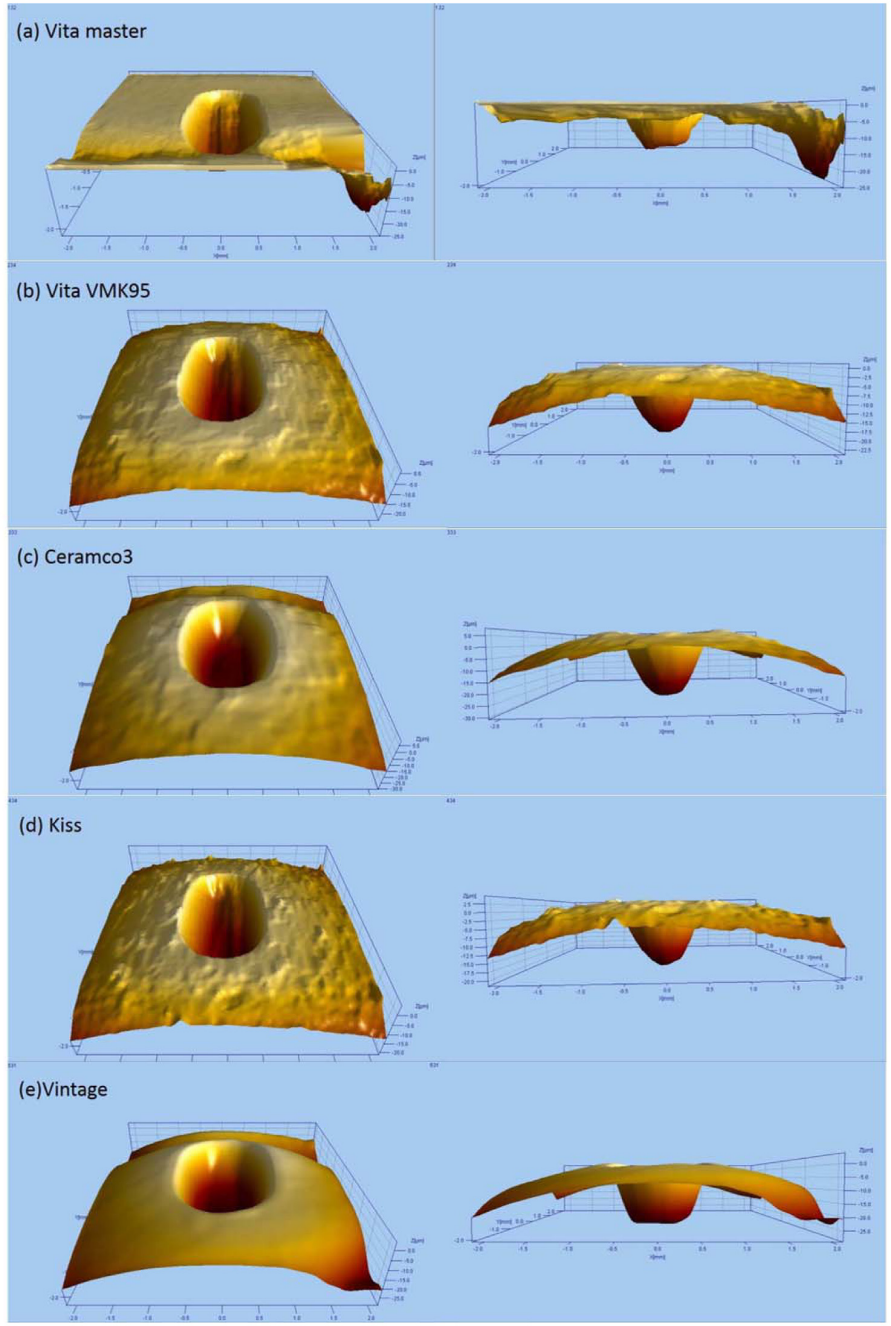
Morphologies of the three-dimensional reconstructions of wear scar under artificial saliva after 10000 cycles. (a) Vita master, (b) Vita VMK95, (c) Ceramco 3, (d) Kiss, and (e) Vintage.

The reconstructed wear scar morphologies are clearly displayed in Fig 3. The shape of wear scar can be divided into two types: oval with characteristic scratch and smooth trapezoidal. The wear surfaces of Vita master, Vita VMK95, and Kiss belong to the first type, as seen in their long oval-shaped concave defects. Although the wear scars of Ceramco 3 and Vintage belong to the second type, the reconstructed wear morphologies reveal that all of the wear scars are shallow around the edges and deep at the center. The vertical sections of Vita master and Vintage are trapezoidal in shape, whereas those of VMK95, Ceramco 3, and Kiss are oval.

### 3.3 Friction coefficient curves with the cycles

**Fig 4:**
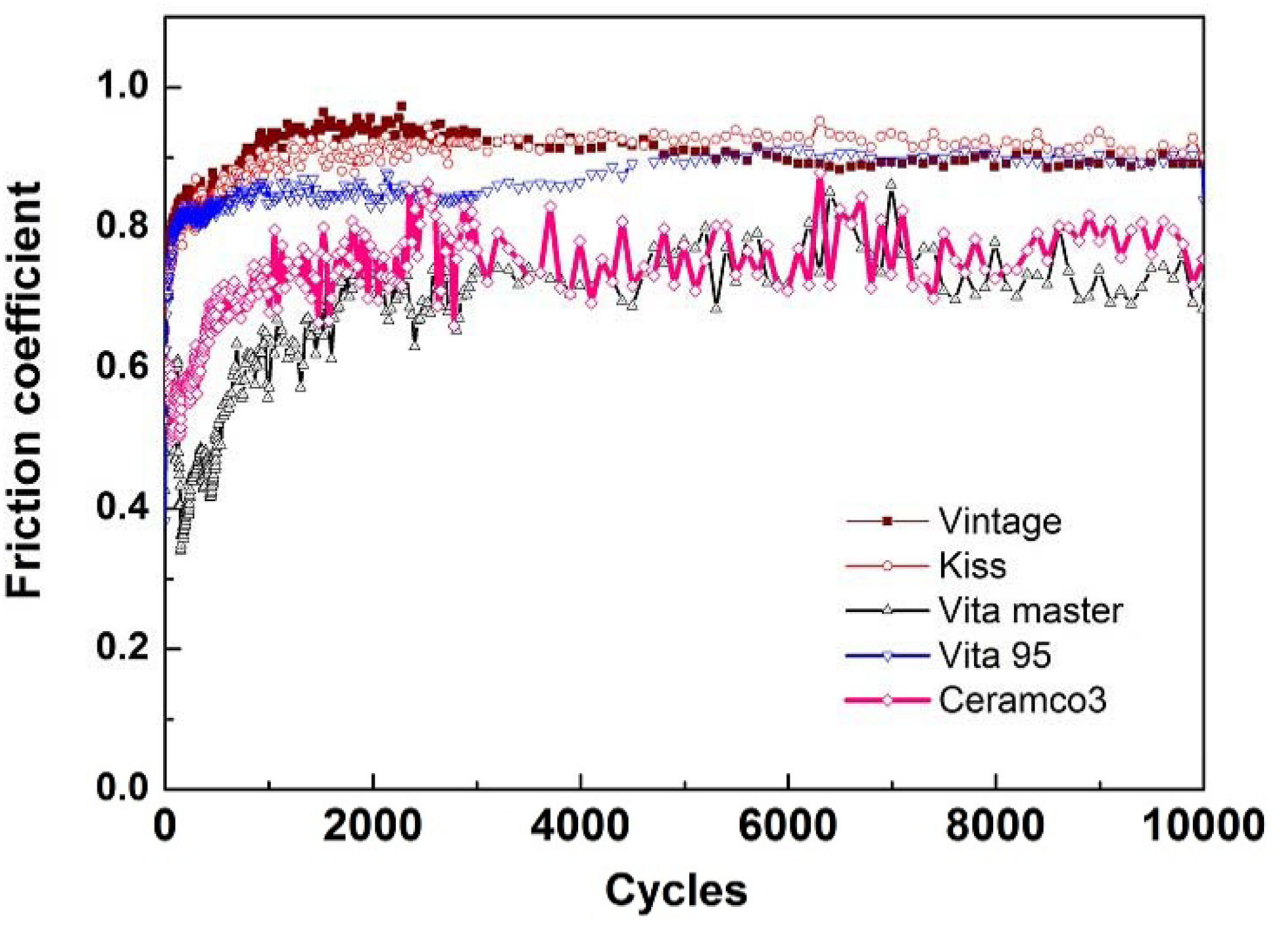
Friction coefficients of five dental veneering porcelains under artificial saliva environment.

The friction coefficients of the five veneering porcelains under saliva lubrication are shown in Fig 4 and S2 Dataset. Friction coefficients can be grouped based on differing behavior in two stages. At the initial stage of testing (before 3000 cycles), the friction coefficients varied greatly prior to achieving relative stability. At the second stage, the friction coefficients remained in steady state. For the five porcelains, curves for friction coefficient are also classified into two categories. In the first type, the friction curve increases slowly with increasing number of cycles during the entire test (i.e., Vita VMK95, Kiss, and Vintage). By contrast, the second type shows sudden increase at the initial stage (i.e., Vita master and Ceramco 3). The steady friction coefficient of Kiss, Vita VMK95, and Vintage is about 0.9, whereas that of Vita master and Ceramco 3 is about 0.7.

### 3.4 Morphologies of wear scar

**Fig 5:**
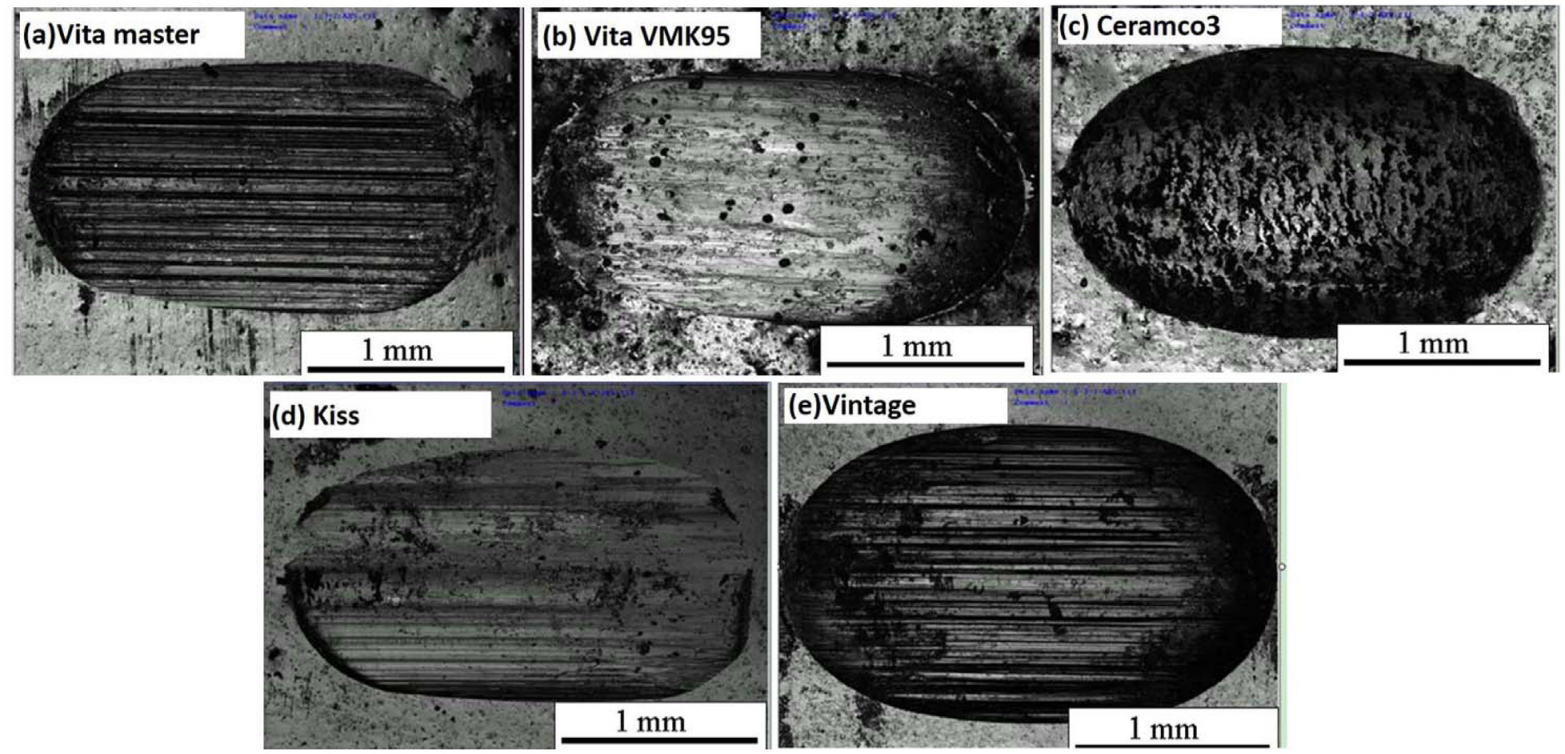
Wear morphologies of dental veneering porcelains under artificial saliva after 10000 cycles. (a) Vita master, (b) Vita VMK95, (c) Ceramco 3, (d) Kiss, and (e) Vintage.

Several types of wear scar were observed under the CLSM. After 10 thousand cycles, the main wear features of all specimens are of two kinds: plough and delamination. The wear surface of Vita master and Vintage prominently exhibit dense plough (Fig 5a and 5e). Vintage possesses more abrasive grains than that found on the surface of Vita master. The wear surface of Vita VMK95 exhibit less plough but reveal some small and abrasive grains (Fig 5b). The wear morphology of Ceramco 3 displays massive exfoliation and no plough (Fig 5c). Cracks and wear particles cover the entire worn surface. Cracks also interweaved, which resulted in the delamination of wear particles. Kiss shows a clear, irregularly distributed plough and some abrasive grains (Fig 5d). Vita master and Vintage predominantly show plough, whereas Ceramco 3 mainly displays delamination. Notably, the damage features are closely related to the microstructure of the materials.

## Discussion

As observed in the experiment, wear resistance capability is associated with the content, size, and distribution of the leucite crystals, as well as the microstructure of the glass matrix.

In our research, the porcelains with higher leucite content presented with higher wear resistance. Vita master was noted with higher concentrations of crystals (Fig 1a) and greater wear resistance. By contrast, Ceramco 3 exhibited lower concentrations of leucite (Fig 1i) and lower capabilities of wear resistance. The ability of a porcelain to resist crack propagation is related to the leucite crystal content. The strength of leucite crystal is higher than that of glass matrix; thus, higher concentrations of crystal will arrest or deflect cracks in glass matrix [14]. Higher crystal content will increase the wear resistance of porcelains [14]. Additionally, previous studies report that higher quantities of leucite (from 10% to 30%) lead to a significant increase in the flexural strength of dental porcelains (from 34.1 MPa to 64.8 MPa) [15, 16]. On the contrary, the study of Kon et al. showed that higher quantities of leucite significantly reduced the mechanical properties of dental porcelains [17]. Therefore, further investigations are needed to explain the quantitative relation between the content of leucite and wear resistance.

Leucite crystal size influences friction and wear behavior. Differently sized leucite crystals may possess different initial microcrack densities, leading to varying friction behavior [18, 19]. In this experiment, the sizes of Ceramco 3 crystals were larger than the other evaluated groups. As displayed in Fig 1k, sharp edges at the interface were exposed after the large crystal was detached from the glass matrix. Previous studies showed that in crystal sizes smaller than 4 μm, microcracks are limited to the crystal. [20, 21]. The excellent wear resistance exhibited by Vita master was influenced by the size of the crystal (approximately 3 μm), which limited the cracks to propagate out of the crystal. Kiss (3–5 μm) and Vita VMK95 (5 μm) exhibited similar wear features. The crystal sizes of Vintage were measured at 1–3 μm, but the material displayed weaker wear resistance that is probably due to the stiffness of glass matrix. By contrast, Ceramco 3 possesses the largest crystal sizes (5–7 μm) with the weakest wear resistance.

The distribution of leucite crystals along the glass matrix influences the strength of porcelains [18], which affects the friction and wear response. As shown in the SEM images, Vita master exhibited a uniform distribution of crystals and displayed excellent wear resistance. The structure of glass matrix also influenced wear resistance. Vintage dental porcelain showed small crystal sizes and uniform distribution but poor wear resistance; these properties may be attributed to the presence of microcracks in the matrix, which can be observed in Fig 1g.

The shape of the crystals did not exhibit a strong effect on wear resistance. The crystal shape of Kiss, Vita master, Vita VMK95, and Ceramco 3 is either circular or oval, whereas that of Vintage is needle-shaped (Fig 1).

**Fig 6:**
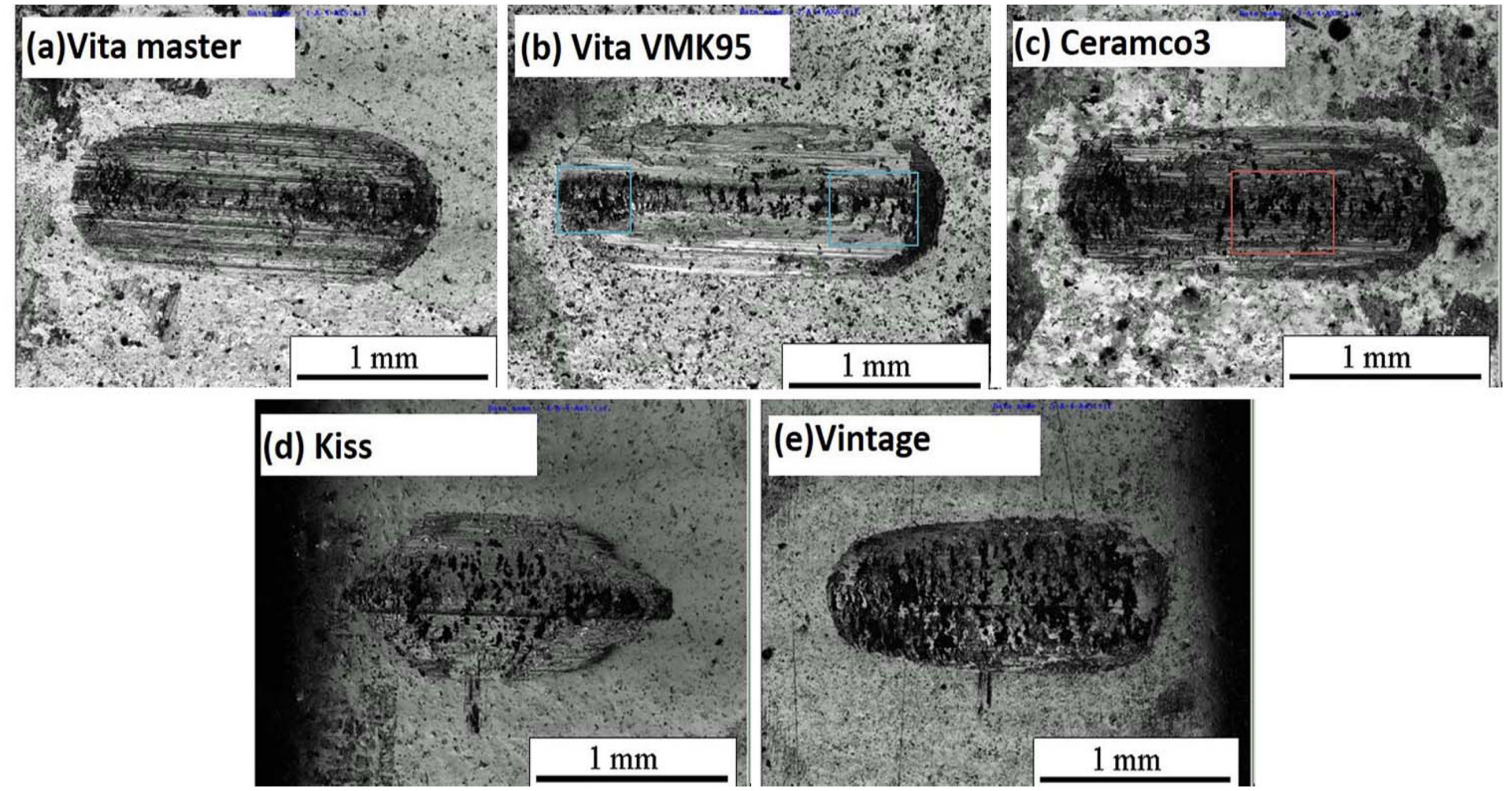
Wear morphologies of dental veneering porcelains under artificial saliva after 2000 cycles. (a) Vita master, (b) Vita VMK95, (c) Ceramco 3, (d) Kiss, and (e) Vintage.

Abrasive wear is a type of damage caused by friction between surfaces containing small hard bumps or hard particles in contact or in relative movement [22]. The main evidence for abrasive wear is plough [23] and is clearly shown in Fig 5 for all specimens at 10000 cycles. The mechanism at early stage was mainly attributed to abrasive wear. For samples over 2000 cycles, the existence of microcracks promoted further crack propagation and delamination that occurred at the later stage (Fig 6). The direction of cracks is perpendicular to the direction of motion because of shear stress [24–26]. From the wear morphology of dental veneering porcelains under artificial saliva after 2000 cycles, two types of cracks were observed: radial and irregular cracks on either side of the wear scar (Fig 6b, blue rectangle); and concentric and irregular cracks at the center of the wear scar (Fig 6c, red rectangle).

For cycles fewer than 10000, the primary damaging mechanism was abrasive wear and delamination (Fig 5). Nucleation in the subsurface, as well as microcrack propagation and intersection, resulted in delamination [15] and consequent exposure of larger and deeply located substance. Wear feature is related to the size and distribution of crystals. Wear scar shows plough when crystal sizes are smaller, e.g., Vita master (3 μm) and Vintage (1–3 μm). The smaller the crystal size, the deeper and denser the plough becomes. In larger crystal sizes, wear scar shows delamination, as seen in Ceramco 3 (5–7 μm). Both plough and delamination are seen for crystal sizes between 3–5 μm, e.g., Kiss and Vita VMK95.

The five different porcelains generated two types of friction coefficient curves. The first type (Kiss, Vita VMK95, and Vintage) showed a slowly increasing trend in friction coefficient with increasing number of cycles. The entire process was steadily abrasive with no fractures seen in the cracking zone. The second type showed a sharp increase in trend of the friction coefficient in the initial stage of the process (Ceramco 3 and Vita master). The abrupt increase in friction coefficient may be attributed to the intense wear in some cycles, which resulted in the corresponding increase in the amount of wear debris [27]. The coefficient of friction subsequently stabilized after the abrupt increase, owing to the process of two-body wear from the formation of microcracks under the area of contact. Given that microcracks were produced on the surface of the veneering porcelain, propagation into net-shaped cracks occurred, and delamination resulted. The process will further progress to three-body wear [28]. Besides, the steady friction coefficient of Vita master and Ceramco 3 was lower than that of Kiss, Vita VMK95, and Vintage. Friction coefficient describes the ratio of the force of friction between two bodies and the force pressing them together. It reflected the intrinsic interaction characteristics of tribology. And it was affected by the imposed load force, surface toughness and mechanical feature of the materials. In this study, the difference of the microstructure which leads to different surface toughness and mechanical feature is the reason for the range of the friction coefficient in the five dental porcelains.

The findings of this research indicate that the wear volume of dental veneering porcelain has no direct relationship to hardness (*r* = 0.2948, *P* > 0.05). Wear volume referred to the volume of the abrasion loss of the material in the process of friction. It reflected the wear resistance of materials. In general, the more amount of wear volume, the worse wear resistance of the material. In this study, the mean wear volumes of Ceramco 3 and Vintage are almost twice that of Vita VMK95 and Kiss, whereas Vita master shows the smallest wear volume. Therefore, Vita master shows better wear resistance than the other porcelains. The five dental porcelains show different mean wear volume, which represents different wear resistance of dental materials. Researches showed that wear process appears to be more closely related to ceramic microstructure, the roughness of contacting surfaces, and lubrication environment rather than hardness [3, 29]. The results of the present study are consistent with such findings.

Dental veneering porcelains are commonly used in porcelain-fused-to-metal and all-ceramic restorations. Based on our findings, friction and wear performance are dependent on crystal content, size, and distribution in glass matrix. When choosing a compatible wear-resistant restoration and exploring new porcelain materials, traditional hardness should not be the main reference factor. High content, small size, uniform crystal distribution, and dense matrix are among the factors that must be considered.

## Conclusions

The friction and wear behavior of dental porcelains with different microstructures were examined. As a result, the following conclusions were obtained:

1. The wear resistance of veneering porcelains is associated with the content, size, and distribution of leucite crystals, as well as the microstructure of the glass matrix, but not with crystal shape. The porcelains with high content, small size, uniform crystal distribution and dense matrix exhibit enhanced wear resistance.
2. Wear mechanism is influenced by crystal size. As for the mechanism of damage, abrasive wear predominates for crystal sizes smaller than 5 μm, whereas delamination is more frequently seen in larger crystal sizes.
3. The wear resistance of the five dental veneering porcelains has no direct relationship with hardness.

## Supporting Information

S1 Dataset Hardness and wear volume of five kinds of dental veneering porcelains. (XLSX)

S2 Dataset Friction coefficients of five dental veneering porcelains under artificial saliva environment. (XLSX)
